# Furostanol Saponins from *Asparagus cochinchinensis* and Their Cytotoxicity

**DOI:** 10.1007/s13659-021-00321-0

**Published:** 2021-11-05

**Authors:** Ruo-Song Zhang, Yang-Yang Liu, Pei-Feng Zhu, Qiong Jin, Zhi Dai, Xiao-Dong Luo

**Affiliations:** 1 State Key Laboratory of Phytochemistry and Plant Resources in West China and Yunnan Key Laboratory of Natural Medicinal Chemistry, Kunming Institute of Botany, Chinese Academy of Sciences, Kunming, 650201 China; 2 University of Chinese Academy of Sciences, Beijing, 100049 China; 3 Key Laboratory of Medicinal Chemistry for Natural Resource, Ministry of Education, Yunnan Provincial Center for Research & Development of Natural Products, School of Chemical Science and Technology, Yunnan University, Kunming, China

**Keywords:** Steroid saponins, *Asparagus cochinchinensis*, Cytotoxicity, Structural elucidation

## Abstract

Phytochemical investigation on the roots of *Asparagus cochinchinensis* led to the isolation of one new furostanol saponin, named 26-*O*-*β*-d-glucopyranosyl-22*α*-hydroxyl-(25*R*)-Δ^5(6)^-furost-3*β*,26-diol-3-*O*-*α*-l-rhamnopyranosyl-(1 → 2)-[*β*-d-glucopyranosyl-(1 → 4)-*α*-l-rhamnopyranosyl-(1 → 4)]-*β*-d-glucopyranoside (**1**), along with three known congeners (**2**‒**4**). The structure of new saponin was elucidated via comprehensive inspection of its HRMS and NMR spectral data as well as chemical technology, whereas those of known ones were identified by comparison of their NMR and MS spectral data with those reported in literatures. All isolated saponins were evaluated for their cytotoxic effects on two human liver (MHCC97H) and lung adenocarcinoma (H1299) cancer cells in vitro. Among them, both **1** and **2** showed significant cytotoxicity against above mentioned cell lines. Further studies revealed that these two saponins could significantly inhibit their proliferation of MHCC97H and H1299 cells.

**Supplementary Information:**

The online version contains supplementary material available at 10.1007/s13659-021-00321-0.

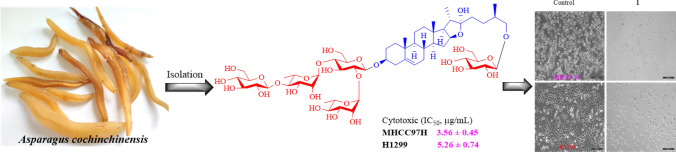

## Introduction

Steroid saponins, whose aglycones were usually a spirostanol or its derivatives [1], were commonly found from roots, tubers, leaves, blooms or seeds in more than 100 families of plants [2, 3]. Compared with other glycosides, the strong foam-forming property in aqueous solution of steroidal saponins was their main feature [2, 4]. Previous researches revealed steroidal saponins possessed various pharmacological activities, such as antifungal [5], hypocholesterolemic [6], antimitotic [7] and cAMP phosphodiesterase inhibitory [8] effects. Among them, a large number of publications have revealed steroid saponins shared different cytotoxic properties that promoted their potential as anti-cancer drugs or adjuvants [9, 10].

**Fig. 1:**
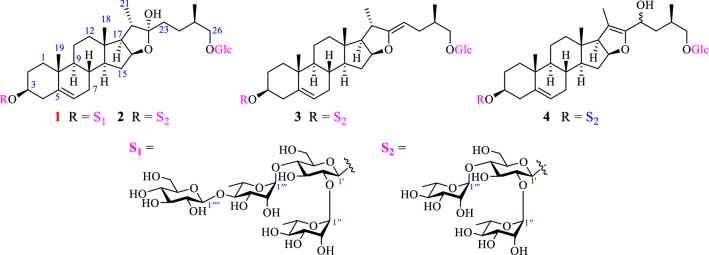
Structures of **1**‒**4**

*Asparagus cochinchinensis*, belonging to the genus *Asparagus* (Liliaceae), is well-known as “Tianmendong” in China. Its roots have been historically used in Chinese folk medicine for the treatment of cough, acute and chronic bronchitis, chronic pharyngitis, hemorrhoids, and tumors for thousands of years [11]. Apart from steroidal saponins [12], phenolic compounds [13], norlignans [14] and alkaloids [15] have been isolated from this plant as revealed by previous phytochemical studies. However, steroidal saponins obtained from title species were proved to be its major and bioactive components responsible for its cytotoxic [16], anti-inflammatory [17], hepatotoxic and nephrotoxic [18], and anti-neuroinflammatory [11] properties. In continuation of a search for bioactive constituents from plants of the Yunnan province [19], a chemical investigation was performed on the roots of *A. cochinchinens*. As a result, a total of steroidal saponins (**1**‒**4**) were isolated and identified including one new and three previously described furostan-type steroidal saponins. Their cytotoxic effects on two human cancer cells MHCC97H and H1299 were also evaluated (Fig. 1).

## Results and Discussion

**Fig. 2:**
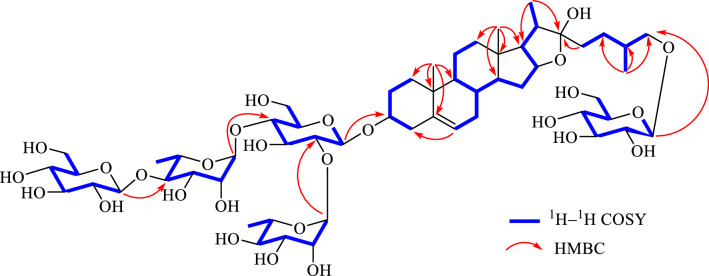
Key ^1^H ‒ ^1^H COSY and HMBC correlations of **1**

**Fig. 3:**
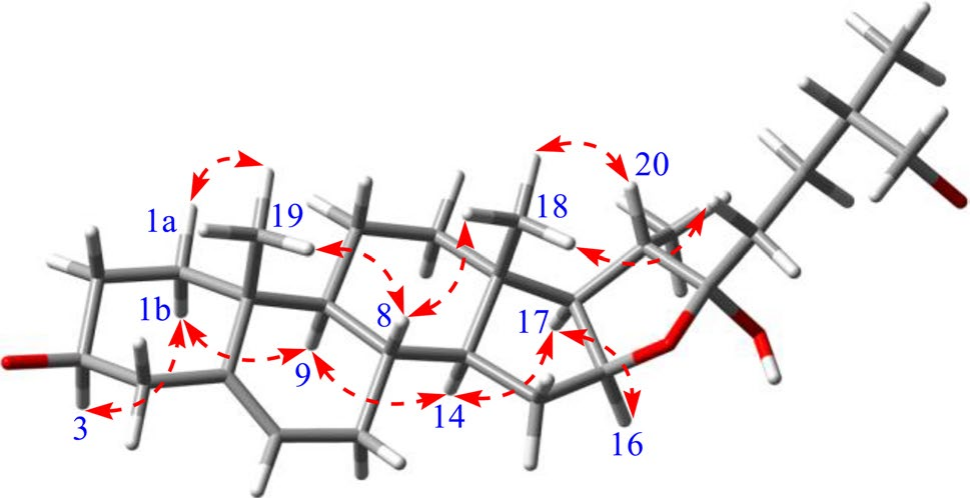
Key ROESY correlations for the aglycone moiety of **1**

Saponin **1** was obtained as a white amorphous powder. It had a molecular formula of C_57_H_94_O_27_ as determined by the observed (+)-HRESIMS protonated ion peak at *m*/*z* 1233.5879 [M + Na]^+^ (calcd for C_57_H_94_O_27_Na, 1233.5875). It showed a positive reaction to the Ehrlich’s reagent (red color), suggesting a furostanol skeleton [20]. The ^13^C NMR spectrum (Table 1) displayed 57 carbons, of which 27 were assigned to the aglycone part and the remaining 30 were attributed to five hexose units. With the aid of the HSQC experiment, the ^1^H and ^13^C NMR spectrum (Table 1) attributable to the aglycone moiety showed resonances for four characteristic steroidal methyls at *δ*_H_ 0.83 (3H, s, CH_3_-19), 0.93 (3H, d, *J* = 6.6 Hz, CH_3_-27), 1.00 (3H, s, CH_3_-18), and 1.26 (3H, d, *J* = 6.7 Hz, CH_3_-21), together with their corresponding carbons at *δ*_C_ 16.3 (CH_3_-19), 17.3 (CH_3_-27), 19.2 (CH_3_-18), 16.3 (CH_3_-21); two oxygenated methines at *δ*_H_ 3.82 (1H, m) and 4.88 (1H, m), along with their corresponding carbons at *δ*_C_ 77.8 (CH-3) and 80.8 (CH-16); an olefinic group at *δ*_H_ 5.26 (1H, brs) as well as *δ*_C_ 121.6 (CH-6) and 140.6 (C-5); and a ketal carbon at *δ*_C_ 110.4 (C-22). The abovementioned data indicated that the aglycone of **1** should be a furostanol one as that of protodioscin (**2**) [21]. Moreover, the aglycone of **1** was further confirmed a by the following diagnostic ^1^H‒^1^H COSY, HMBC, and ROESY correlations (Figs. 2 and 3). The ^1^H‒^1^H COSY experiment revealed three structural fragments including CH_2_-1‒CH_2_-2-CH-3‒CH_2_-4, CH-6‒CH_2_-7‒CH-8/(‒CH-9‒CH_2_-11‒CH_2_-12)/‒CH-14‒CH_2_-15‒CH-16‒CH-17‒CH-20‒CH_3_-21, and CH_2_-23‒CH_2_-24‒CH-25/(‒CH_3_-27)/‒CH_2_-26. Moreover, the observed HMBC from *δ*_H_ 1.00 (CH_3_-18) to *δ*_C_ 39.7 (CH_2_-12), 40.4 (C-13), 56.4 (CH-14), and 63.6 (CH-17), from *δ*_H_ 0.83 (CH_3_-19) to *δ*_C_ 37.3 (CH_2_-1), 140.6 (C-5), 50.1 (CH-9), and 36.9 (C-10), and from both *δ*_H_ 1.26 (CH_3_-21) and *δ*_H_ 2.00 (H-23a) to *δ*_C_ 110.4 (C-22) established the aglycone of **1** to be 22*α*-hydroxyl-(25*R*)-furost-Δ^5(6)^-3*β*,26-diol. The ROESY correlations of *δ*_H_ 1.00 (Me-18) with 1.51 (H-8)/2.17 (H-20)/1.94 (H-23b) and of *δ*_H_ 0.83 (Me-19) with 1.51 (H-8) and 1.68 (H-1a) verified these protons were placed at the same side, whereas the observed ROESY correlations of *δ*_H_ 0.94 (H-1b) with 3.82 (H-3)/0.86 (H-9), of *δ*_H_ 1.02 (H-14) with 0.86 (H-9)/1.87 (H-17), and of *δ*_H_ 1.87 (H-17) with 4.88 (H-16) indicated these protons were located at the other side. Additionally, the 25*R* configuration of **1** was assigned according to the small chemical shift difference between Ha-26 and Hb-26 at Δab = 0.34 ppm (Δab > 0.57 ppm for 25*S*, and Δab < 0.48 ppm for 25*R*) [22]. In view of aforementioned evidence, the aglycone of **1** was thus elucidated as 22*α*-hydroxyl-(25*R*)-furost-Δ^5(6)^-3*β*,26-diol.

**Table 1:** ^1^H and ^13^C NMR spectral data of **1** (600 and 150 MHz, pyridine-*d*_5_)

No	Aglycone moiety		No	Sugar moiety	
	*δ*_C_	*δ*_H_ (mult., *J*)		*δ*_C_	*δ*_H_ (mult., *J*)
1	37.3, CH_2_	a 1.68 m b 0.94 m	3-*O*-Glc		
			1′	100.1, CH	4.88 d (7.7)
2	29.9, CH_2_	a 1.98 m b 1.79 m	2′	77.6, CH	4.10 m
3	77.8, CH	3.82 m	3′	73.8, CH	4.26 m
4	38.7, CH_2_	a 2.71 m b 2.64 m	4′	77.1, CH	4.31 m
5	140.6, C		5′	76.7, CH	4.30 m
6	121.6, CH	5.26 br s	6′	61.0, CH_2_	a 4.12 m b 3.98 m
7	32.1, CH_2_	1.83 2H m			
8	31.5, CH	1.51 m	2′-*O*-Rha		
9	50.1, CH	0.86 m	1″	101.6, CH	6.27 br s
10	36.9, C		2″	71.4, CH	4.76 m
11	20.9, CH_2_	1.38 2H m	3″	72.5, CH	4.75 m
12	39.7, CH_2_	a 1.70 m b 1.06 m b1.06 m	4″	73.8, CH	4.26 m
13	40.4, C		5″	69.3, CH	4.84 m
14	56.4, CH	1.02 m	6″	18.4, CH_3_	1.68 3H d (6.0)
15	32.2, CH_2_	1.40 2H m	4′-*O*-Rha		
16	80.8, CH	4.88 m	1‴	101.8, CH	5.74 br s
17	63.6, CH	1.87 m	2‴	71.7, CH	4.76 m
18	19.2, CH_3_	1.00 3H s	3‴	72.2, CH	4.59 m
19	16.3, CH_3_	0.83 3H s	4‴	84.9, CH	4.35 m
20	40.6, CH	2.17 m	5‴	68.3, CH	4.93 m
21	16.3, CH_3_	1.26 3H d (6.7)	6‴	18.2, CH_3_	1.60 3H d (6.0)
22	110.4, C		4″-*O*-Glc		
23	36.9, CH_2_	a 2.00 m b 1.94 m b 1.94 m	1″″	106.4, CH	5.14 d (7.7)
24	28.1, CH_2_	a 1.97 m b 1.63 m	2″″	76.7, CH	3.98 m
25	34.0, CH	1.93 m	3″″	78.2, CH	3.70 m
26	74.9, CH_2_	a 3.55 dd (9.0, 6.1) b 3.88 m	4″″	71.0, CH	4.12 m
27	17.3, CH_3_	0.93 3H d (6.6)	5″″	76.3, CH	4.00 m
			6″″	62.2, CH_2_	a 4.45 d (12.4) b 4.28 m
			26-*O*-Glc		
			1‴″	104.6, CH	4.73 d (7.8)
			2‴″	75.0, CH	3.82 m
			3‴″	78.2, CH	3.99 m
			4‴″	71.7, CH	4.12 m
			5‴″	78.3, CH	4.10 m
			6‴″	62.5, CH_2_	a 4.45 m b 4.28 m

**Scheme 1:**
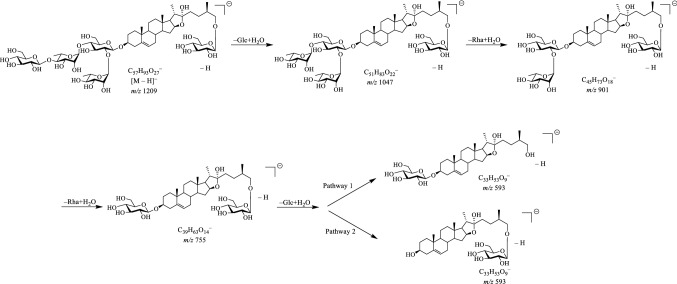
The fragmentation process of **1** in the ESI- MS negative scan

As for the sugar units of **1**, its ^1^H NMR spectrum (Table 1) displayed the presence of five anomeric proton signals at *δ*_H_ 4.73 (1H, d, *J* = 7.8 Hz, H-1‴″), 4.88 (1H, d, *J* = 7.7 Hz, H-1′), 5.14 (1H, d, *J* = 7.7 Hz, H-1″″), 5.74 (1H, brs, H-1‴) and 6.27 (1H, brs, H-1″), which showed correlations in the HSQC spectrum with five anomeric carbons at *δ*_C_ 104.6 (CH-1‴″), 100.1 (CH-1′), 106.4 (CH-1″″), 101.8 (CH-1‴), and 101.6 (CH-1″). With the assistance of MS spectrum, the sugar moiety of **1** was preliminary determined. Specifically, the [M ‒ H]^‒^ ion (*m*/*z* 1209.6) displayed **1** had a molecular weight (MW) of 1210.6 Da in the negative ion mode of ESI-MS^n^. The observed ions with *m*/*z* values of 1047.5, 901.5, and 755.4 indicated the sequential cleavage of two rhamnopyranosyl units followed by the cleavage of a glucopyranosyl moiety from the parent [M ‒ H]^‒^ ion (*m*/*z* 1209.6), respectively. Likewise, the MS^2^ spectrum also afforded *m*/*z* value of 593.4 that was indicative of the loss of one glucopyranosyl group from the C-3 position or the C-26 position (Scheme 1). Also, acid hydrolysis of **1** also gave d-glucoses and l-rhamnoses as the sugar residue, which was confirmed by HPLC analysis of their corresponding PMP derived adducts. All the anomeric protons of d-glucose possessed *β*-configurations due to their ^3^*J*_H1, H2_ coupling constants (7.8, 7.7, and 7.7 Hz), and both anomeric protons of l-rhamnoses shared *α*-configurations due to the chemical shifts of C-3 (*δ*_C_ 72.5 and 72.2) and C-5 (*δ*_C_ 69.3 and 68.3), respectively. In the HMBC spectrum, the long-range correlations from *δ*_H_ 4.88 (H-1′) to *δ*_C_ 77.8 (CH-3), from *δ*_H_ 4.73 (H-1‴″) to *δ*_C_ 74.9 (CH_2_-26), from *δ*_H_ 6.27 (H-1″) to *δ*_C_ 77.6 (CH-2′), from *δ*_H_ 5.74 (H-1‴) to *δ*_C_ 77.1 (CH-4′), and from *δ*_H_ 5.14 (H-1″″) to *δ*_C_ 84.9 (CH-4‴) established the sequence for 3-*O*-sugar chain as an [*α*-l-rhamnopyranosyl-(1 → 2)]-[*β*-d-glucopyranosyl-(1 → 4)-*α*-l-rhamnopyranosyl-(1 → 4)]-*β*-d-glucopyranosyl moiety and for 26-*O*-sugar chain as *β*-d-glucopyranosyl moiety, respectively. Based on the above information presented, the structure of **1** was thus elucidated to be 26-*O*-*β*-d-glucopyranosyl-22*α*-hydroxyl-(25*R*)-Δ^5(6)^-furost-3*β*,26-diol-3-*O*-*α*-l-rhamnopyranosyl-(1 → 2)-[*β*-d-glucopyranosyl-(1 → 4)-*α*-l-rhamnopyranosyl-(1 → 4)]-*β*-d-glucopyranoside.

Additionally, three known steroidal glycosides were identified as protodioscin (**2**) [21], (25*R*)-26-*O*-*β*-d-glucopyranosyl-3*β*,20*α*,26-trihydroxyfurostan-5, 22-diene-3-*O*-*α*-l-rhamnopyranosyl-(1 → 2)-[*α*-L-rhamnopyranosyl-(1 → 4)]-*O*-*β*-d-glucopyranoside (**3**) [23], and dioscoreside H (**4**) [24] by comparison of their spectroscopic data with those reported in the literatures.

**Fig. 4:**
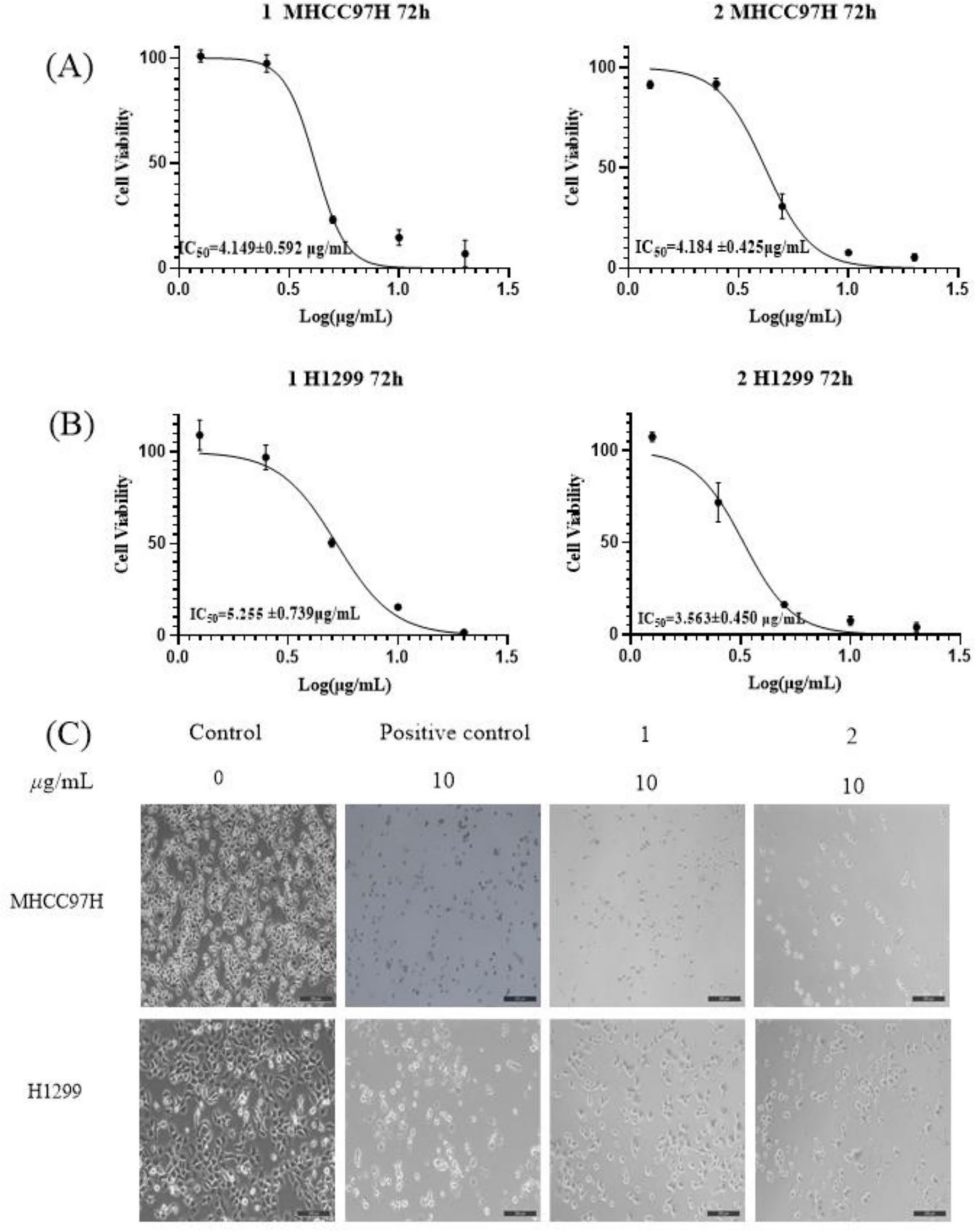
Effects of **1** and **2** on MHCC97H and H1299 cells proliferation (*n* = 3). **A** The IC_50_ values of **1** and **2** against MHCC97H; **B** The IC_50_ values of **1** and **2** against H1299; **C** Inhibition effects of MHCC97H and H1299 cells proliferation by **1** and **2** after cultivation for 72 h

The steroid saponins obtained from species of Liliaceae have shown the potential to significantly inhibit the proliferations of various human tumor cell lines in vitro [25–29]. Therefore, all isolated compounds were evaluated for their cytotoxicity against MHCC97H and H1299 by the MTT method. More specifically, compared with the IC_50_ values of positive control doxorubicin hydrochloride, and both **1** and **2** displayed strong cytotoxicity against MHCC97H and H1299 cells with IC_50_ values of 3.56 ± 0.45/4.18 ± 0.43 μg/mL and 5.26 ± 0.74/4.15 ± 0.59 μg/mL, respectively (see Fig. 4). Furthermore, as can be seen from Fig. 4, compared with the positive control doxorubicin hydrochloride, saponins **1** and **2** could significantly inhibit their proliferation (Table 2).

**Table 2:** Cytotoxicity of saponins **1** and **2** (IC_50_ ± SD, *μ*g/mL)

Compound	H1299	MHCC97H
**1**	5.26 ± 0.74	3.56 ± 0.45
**2**	4.15 ± 0.59	4.18 ± 0.43
Doxorubicin hydrochloride^a^	0.86 ± 0.39	0.20 ± 0.08

^a^Positive control

Moreover, all obtained steroid saponins were evaluated for their antimicrobial activity against *Escherichia coli* (ML-35P), *Bacillus cereus* (CMCC(B) 63303), *Candida albicans* (ATCC 2091), *Bacillus subtilis* (ATCC 6633), *Streptococcus hemolyticus* (ATCC 19615), *Listeria monocytogenes* (ATCC 19114), *Pseudomonas aeruginosa* (PO01), *Staphylococcus aureus* (ATCC 4330), *Salmonella Typhimurium* (SL1344) and *Staphylococcus epidermidis* (CMCC 26069) by the microdilution broth susceptibility assay. The results (see Table 3) revealed that saponins **1**‒**4** showed moderate antimicrobial activity against *C. albicans* and *B. subtilis*, while only saponin **3** showed weak antimicrobial activity against *S. aureus* (63.30 ± 0.55 μg/mL).

**Table 3:** Antimicrobial activity of saponins **1**‒**4** (IC_50_ ± SD, μg/mL)

Compound	*C. albicans*	*B. subtilis*	*S. aureus*
**1**	55.11 ± 0.32	47.93 ± 0.18	NA^a^
**2**	72.05 ± 0.49	69.30 ± 0.16	NA^a^
**3**	52.05 ± 0.31	47.19 ± 0.19	63.30 ± 0.55
**4**	52.05 ± 0.31	30.07 ± 0.22	NA^a^
Streptomycin sulfat^a^	40.88 ± 0.33	93.49 ± 0.50	22.97 ± 0.24

*NA* no activity (> 100 μg/mL)

^a^Positive control

## Experimental

### General Experiment Procedures

Optical rotation was measured on a Autopol VI automatic polarimeter. The IR spectrum were measured on a Thermo Nicolet iS10 infrared spectrophotometer with KBr disk. The NMR spectra were obtained on Bruker DRX-400 and DRX-600 spectrometers. Chemical shifts (*δ*) were expressed in ppm with reference to the solvent signals. Both ESI and HRESIMS spectra were performed on an UPLC-IT-TOF spectrometer. Semi-preparative HPLC was performed on a Waters 600 with a COSMOSIL C18 (10 × 250 mm, Nacalai Tesque Corporation, Japan) column. Analytical HPLC was performed on a Shimadzu SIL-20A Series HPLC system equipped with a reverse-phase COSMOSIL C18 column (4.6 mm × 250 mm, 5 μm, Nacalai Tesque Corporation, Japan). Column chromatography was carried out using silica gel (100‒200 mesh, Qingdao Haiyang Chemical, Qingdao, Co., Ltd., People’s Republic of China) and macro-porous absorption resin (D101, Donghong Chemical Co., Ltd., People’s Republic of China). The PMP (Chengdu Aikeda Chemical Reagent Co., Ltd., China) was purchased from Beijing 4A Biotech Co., Ltd. (Beijing, China). Fractions were monitored by TLC, and spots were visualized by heating silica gel plates sprayed with Ehrlich’s reagent.

### Plant Materials

The roots of *A. cochinchinensis* was purchased from ‘Luosiwan’ Chinese herbal medicine Market, Kunming, Yunnan Province, in November 2019, identified by Dr. Xu-Jie Qin. A voucher specimen (No. Luo 20191106) has been deposited at State Key Laboratory of Phytochemistry and Plant Resource in West China, Kunming Institute of Botany, Chinese Academy of Sciences.

### Extraction and Isolation

The air-dried roots of *A. cochinchinensis* (5.0 kg) were extracted with 90% aqueous EtOH at 80 ℃ (15 L × 4, each time for 3 h). The solvent was removed under reduced pressure to yield an amber residue (2.5 kg). The residue was subjected to column chromatography over an macroporous resin column eluted first with H_2_O then successively with 25%, 70%, and 90% EtOH, respectively. The 70% EtOH partition was evaporated under reduced pressure to obtain a total steroidal saponin moiety. The total saponins (153 g) was subjected to a silica gel column eluting with a CHCl_3_‒MeOH‒H_2_O gradient (80:20:2 → 65:35:10) to yield five fractions (Fr. A‒Fr. E). Fraction C (105 g) was chromatographed on a silica gel column (CHCl_3_‒MeOH‒H_2_O, 9:1:0.1) to give saponin **2** (70 g) and Fr. C1. Fr. C1 (230.5 mg) was further purified by semi-preparative HPLC to afford **1** (29.8 mg; *t*_R_ = 12 min; MeCN‒H_2_O, 28:72, 3.0 mL/min). Fraction D (12 g) was separated on a silica gel column (CHCl_3_‒MeOH‒H_2_O, 8:2:0.2) and then purified by semi-preparative HPLC to yield saponins **3** (3.4 mg, *t*_R_ = 20.5 min; MeCN‒H_2_O, 35:65, 1.0 mL/min) and **4** (2.6 mg, *t*_R_ = 23.5 min; CH_3_CN‒H_2_O, 35:65, 1.0 mL/min).

### Spectroscopic Data of 1

26-*O*-*β*-d-glucopyranosyl-22*α*-hydroxyl-(25*R*)-Δ^5(6)^-furost-3*β*,26-diol-3-*O*-*α*-l-rhamnopyranosyl-(1 → 2)-[*β*-d-glucopyranosyl-(1 → 4)-*α*-l-rhamnopyranosyl-(1 → 4)]-*β*-d-glucopyranoside (**1**): white amorphous powder, [*α*] ‒46.86 (*c* 0.11, MeOH); IR (*ν*_max_): 3417, 2933, 2851, 1635, 1453, 1382, 1045 cm^‒1^; HRESIMS *m*/*z* 1233.5879 [M + Na]^+^ (calcd for C_57_H_94_O_27_Na, 1233.5875). ^1^H (pyridine-*d*_5_, 600 MHz) and ^13^C (pyridine-*d*_5_, 150 MHz) NMR spectral data, see Table 1.

### Acid Hydrolysis of 1

The acid hydrolysis of compound **1** was carried out by a previously reported procedure [19]. Compound **1** (2.0 mg) was refluxed at 120 °C for 2 h with 2 M TFA on an oil bath. The aglycone was removed by the extraction with CHCl_3_ (5.0 mL) for three times. The reaction residue was filtered after neutralizing with 60.0 μL of NaOH (0.3 M). After removing the solvent under reduced pressure, the residue was refluxed at 75 °C for 1 h with 60.0 μL of PMP (0.5 M in methanol). Moreover, the reaction was quenched with 60.0 μL of HCl (0.3 M) and the reaction mixture was extracted with CHCl_3_ (5.0 mL, three times). Then, the aqueous layer was analyzed over HPLC (18% acetonitrile: 82% sodium phosphate (pH 6.8; 1.5 mL/min). Likewise, the standard monosaccharides d-glucose (1.0 mg) and l-rhamnose (1.0 mg) were derivatized with PMP by the same way as **1**, and HPLC analyses were performed under the same conditions as **1**. The sugar units in **1** were identified as d-glucose (*t*_R_ = 14.5 min) and l-rhamnose (*t*_R_ = 17.0 min) by comparison of the retention times of the corresponding derivatives.

### Cytotoxicity Assay

The cytotoxicity of isolated compounds was determined to use the MTT method with a slight modification [30]. Briefly, two human cancer (MHCC97H and H1299) cell lines were incubated in 96-well plates at a density of 2 × 10^3^ cells/well in DMEM medium supplemented with 10% fetal bovine serum at 37 ℃ with 5% CO_2_. After overnight incubation, cells were treated with tested compounds at different concentrations (20.00, 10.00, 5.00, 2.50, and 1.25 μg/mL) for 72 h. Subsequently, the culture mediums were exchanged by DMEM medium which contained 10% MTS reagent [3-(4,5-dimethylthiazol-2-yl)-5-(3-carboxymethoxyphenyl)-2-(4-sulfophenyl)-2H-tetrazolium, inner salt] and then cultured for another 4 h. The absorbance was recorded on a microplate reader at 490 nm.

### Antimicrobial Activity Assay

The antimicrobial activity of isolated steroid saponins against 10 strains using the microdilution broth susceptibility assay [31]. The strains frozen in the refrigerator at ‒ 80 ℃ were activated and inoculated on standard tryptone soy broth agar (TSA) plates at 37 ℃ for 8 h to observe the bacterial growth. Subsequently, single colonies were selected and inoculated in tryptone soy broth (TSB) plates. After cultivated at 37 ℃ in shaker (120 rpm) for 8 h, the absorbance of bacterial solution was measured and its concentration was adjusted to 10^5^ CFU/mL. Whereafter, an inoculum of 10^5^ CFU/mL was made to sterile 96-well plate containing tested compounds at different concentrations (100.00, 50.00, 25.00, 12.50, 6.25 and 3.13 μg/mL) at 37 ℃ for 8 h. The wells containing only broth served as growth control. The absorbance of bacterial solution was recorded on a microplate reader at 600 nm.

## Conclusion

In summary, a chemical examination of the roots of *A. cochinchinensis* led to the identification of one new furostanol glycoside 26-*O*-*β*-d-glucopyranosyl-22*α*-hydroxyl-(25*R*)-Δ^5(6)^-furost-3*β*,26-diol-3-*O*-*α*-l-rhamnopyranosyl-(1 → 2)-[*β*-d-glucopyranosyl-(1 → 4)-*α*-l-rhamnopyranosyl-(1 → 4)]-*β*-d-glucopyranoside (**1**) and three known one (**2**‒**4**). Meanwhile, compounds **1** and **2** exhibited cytotoxic and anti-proliferative effects on two human (MHCC97H and H1299) cancer cell lines. At the same time, compounds **1**‒**4** displayed moderate antimicrobial activity against *C. albicans* and *B. subtilis*, and compound **3** displayed weak antimicrobial activity against *S. aureus*.

## Supplementary Information

Below is the link to the electronic supplementary material.

Supplementary file1 (DOCX 6762 kb)
