# Supplementary material for: Furostanol Saponins from Asparagus cochinchinensis and Their Cytotoxicity
Source: Nat Prod Bioprospect. 2021 Nov 5;11(6):651–8. doi: 10.1007/s13659-021-00321-0 (PMC8599559; doi:10.1007/s13659-021-00321-0)

Supplementary data for

**Furostanol Saponins from *Asparagus cochinchinensis* and Their Cytotoxicity**

**Ruo-Song Zhang^1,2^**, **Yang-Yang Liu^3^**, **Pei-Feng Zhu^1,2^**, **Qiong Jin^1,2^**, **Zhi Dai^3^**, **Xiao-Dong Luo^1,3^,***

*^1^ State Key Laboratory of Phytochemistry and Plant Resources in West China and Yunnan Key Laboratory of Natural Medicinal Chemistry, Kunming Institute of Botany, Chinese Academy of Sciences, Kunming 650201, China*

*^2^ University of Chinese Academy of Sciences, Beijing 100049, China*

*^3^ Key Laboratory of Medicinal Chemistry for Natural Resource, Ministry of Education; Yunnan Provincial Center for Research & Development of Natural Products; School of Chemical Science and Technology, Yunnan University*

** Corresponding author*

*E-mail address:* xdluo@mail.kib.ac.cn

**Contents**

**Figure S1.** ^1^H NMR Spectrum (600 MHz, pyridine-*d*_5_) of compound **1**1

**Figure S2.** ^13^C NMR Spectrum (150 MHz, pyridine-*d*_5_) of compound **1** 1

**Figure S3.** HSQC Spectrum of compound **1** 2

**Figure S4.** HMBC Spectrum of compound **1**2

**Figure S5.** ^1^H-^1^H COSY Spectrum of compound **1** 3

**Figure S6.** ROESY Spectrum of compound **1** 3

**Figure S7.** HRESIMS Spectrum of compound **1** 4

**Figure S8.** IR Spectrum of compound **1** 5

**Figure S9.** ESI-MS^n^ Spectrum of compound **1** 6-7

**Figure S1.** ^1^H NMR Spectrum (600 MHz, pyridine-*d_5_*) of compound **1**

**Figure S2.** ^13^C NMR Spectrum (150 MHz, pyridine-*d_5_*) of compound **1**

**Figure S3.** HSQC Spectrum of compound **1**

**Figure S4.** HMBC Spectrum of compound **1**

**Figure S5.** ^1^H-^1^H COSY Spectrum of compound **1**

**Figure S6.** ROESY Spectrum of compound **1**

**Figure**
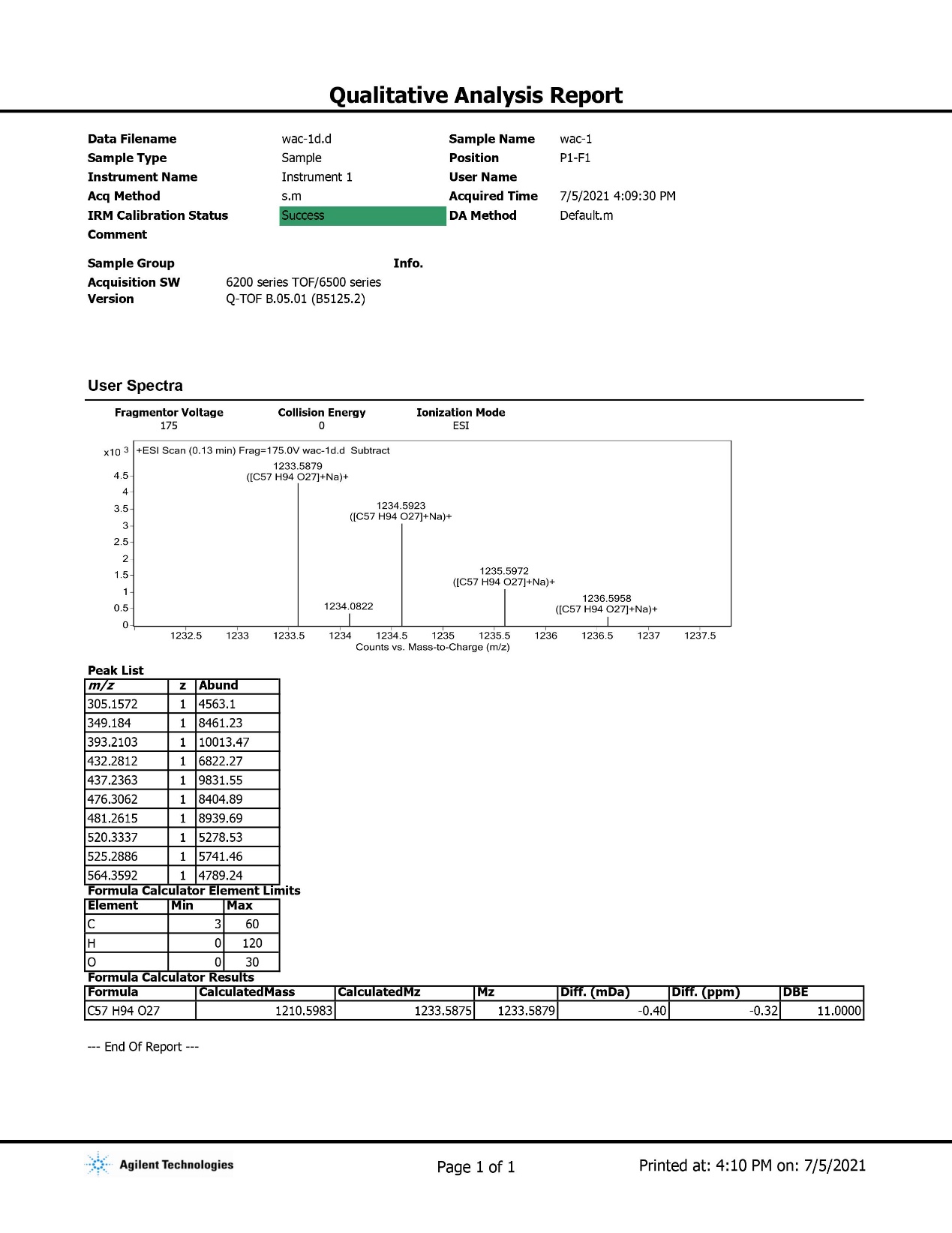
**S7.** HRESIMS Spectrum of compound **1**

**Figure S8.** IR Spectrum of compound **1**

**Figure
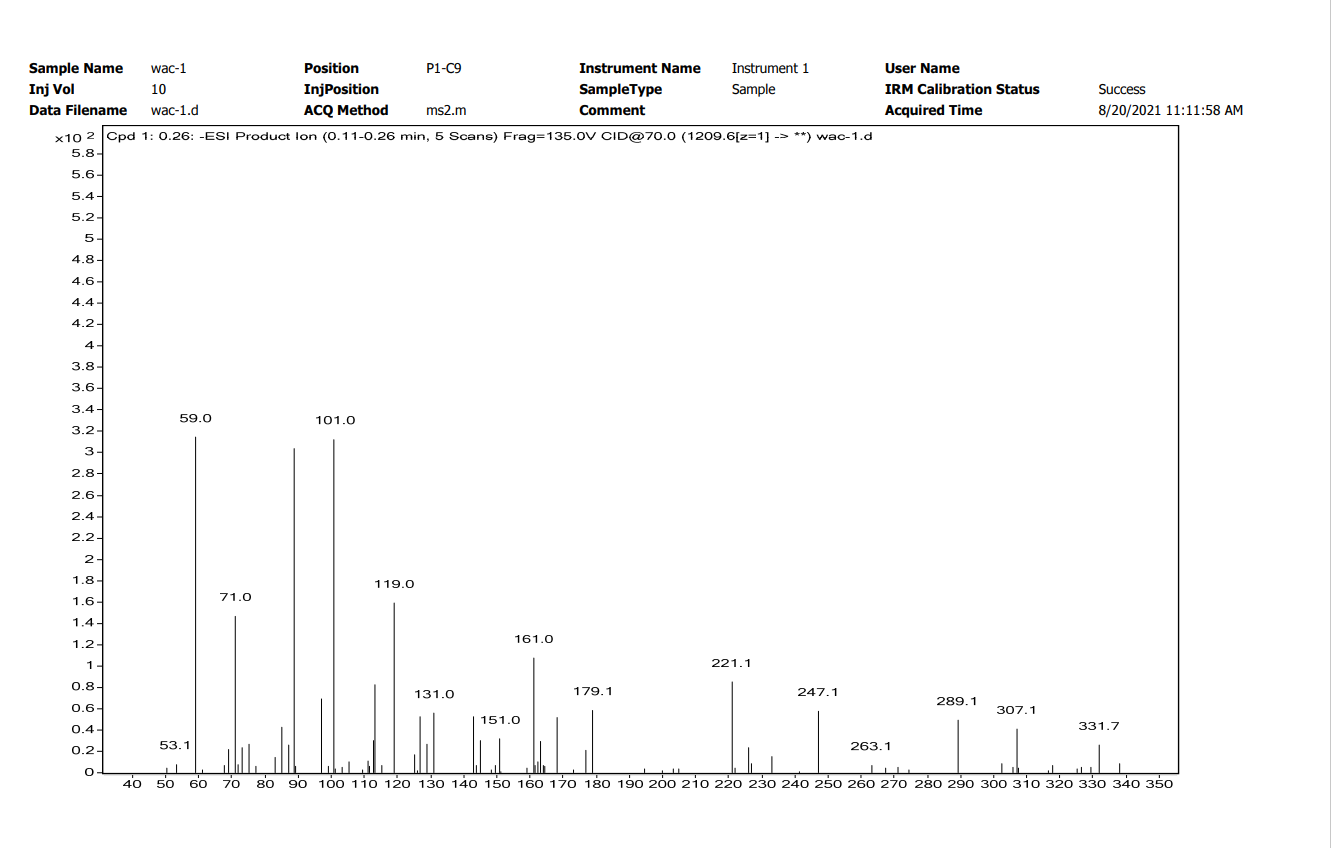
****S9.** ESI-MS^n^ Spectrum of compound **1**


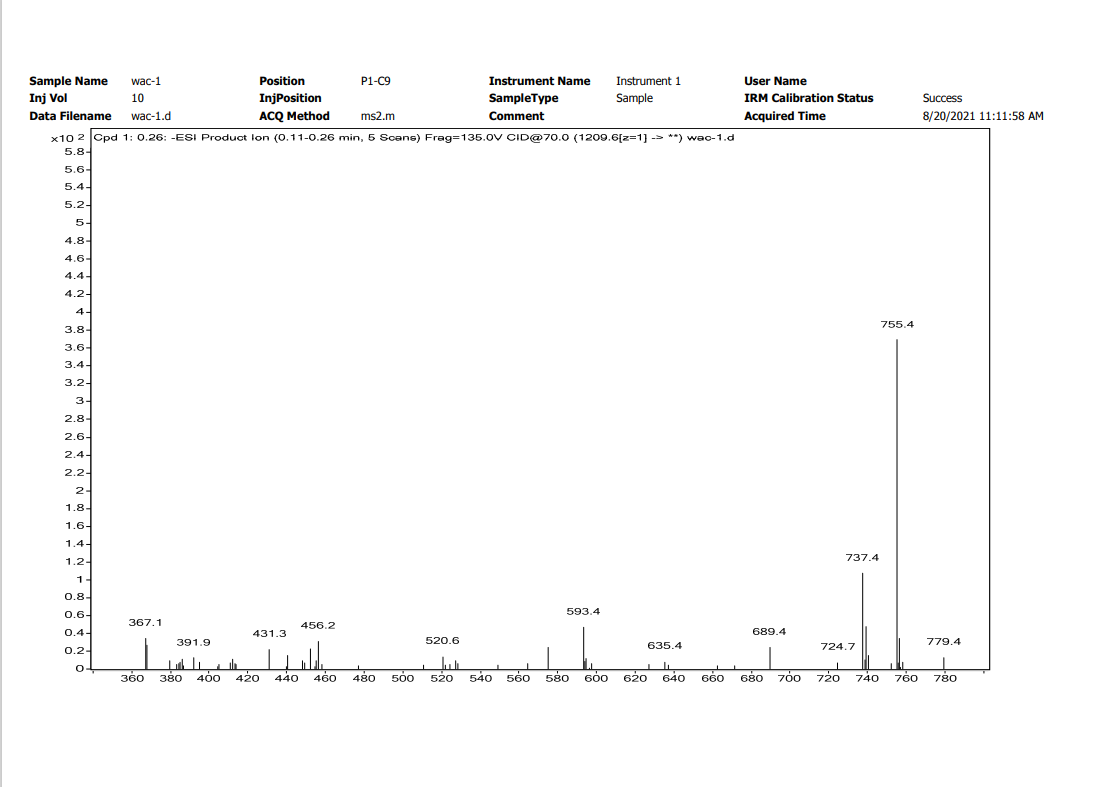


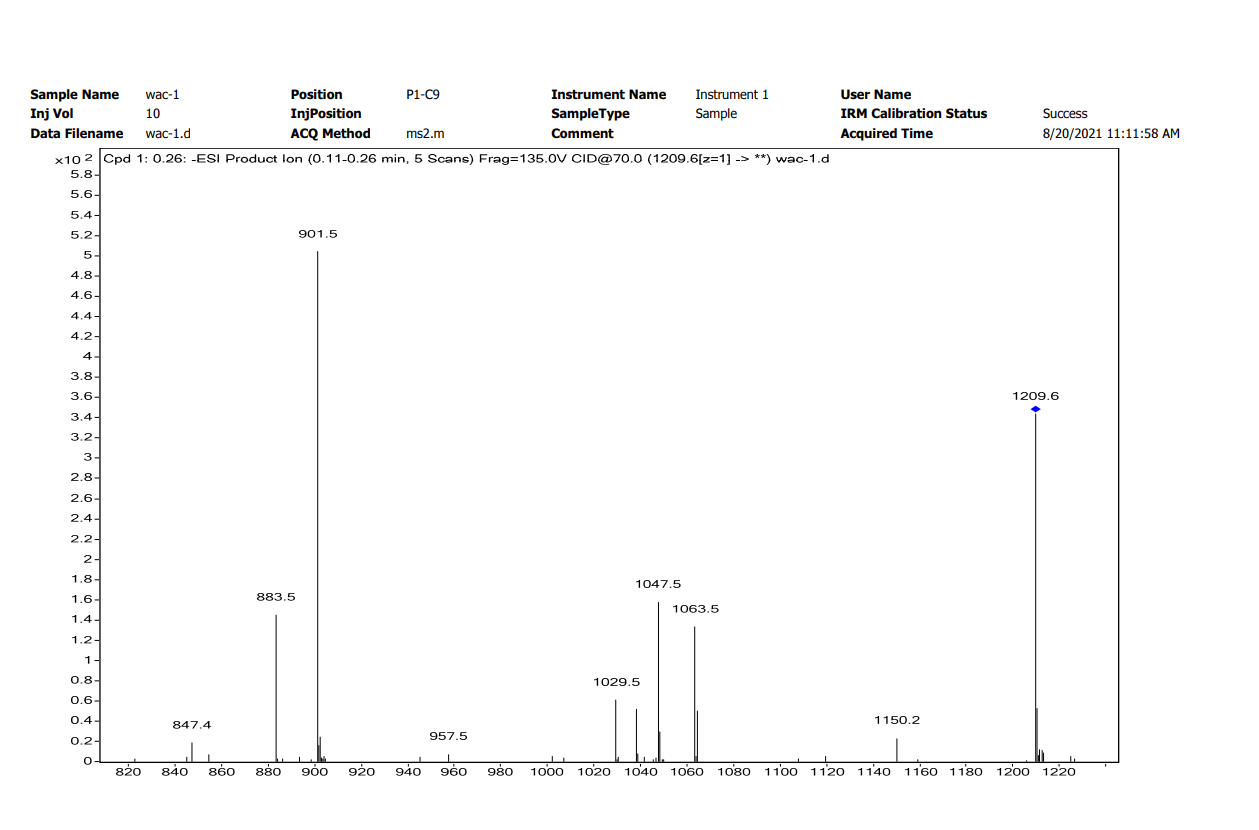

Supplement: Supplementary file 1 — Supplementary file1 (DOCX 6762 kb) [file 13659_2021_321_MOESM1_ESM.docx]
